# Ultrasensitive Photothermal Switching with Resonant Silicon Metasurfaces at Visible Bands

**DOI:** 10.1021/acs.nanolett.3c03288

**Published:** 2023-11-16

**Authors:** Ying Che, Tianyue Zhang, Tan Shi, Zi-Lan Deng, Yaoyu Cao, Bai-Ou Guan, Xiangping Li

**Affiliations:** † Guangdong Provincial Key Laboratory of Optical Fiber Sensing and Communications, Institute of Photonics Technology, Jinan University, Guangzhou 510632, China; ‡ State Key Laboratory of Information Photonics and Optical Communications & School of Integrated Circuits, Beijing University of Posts and Telecommunications, Beijing 100876, China

**Keywords:** bound states in the continuum, photothermal, silicon metasurfaces, ultrasensitive switching

## Abstract

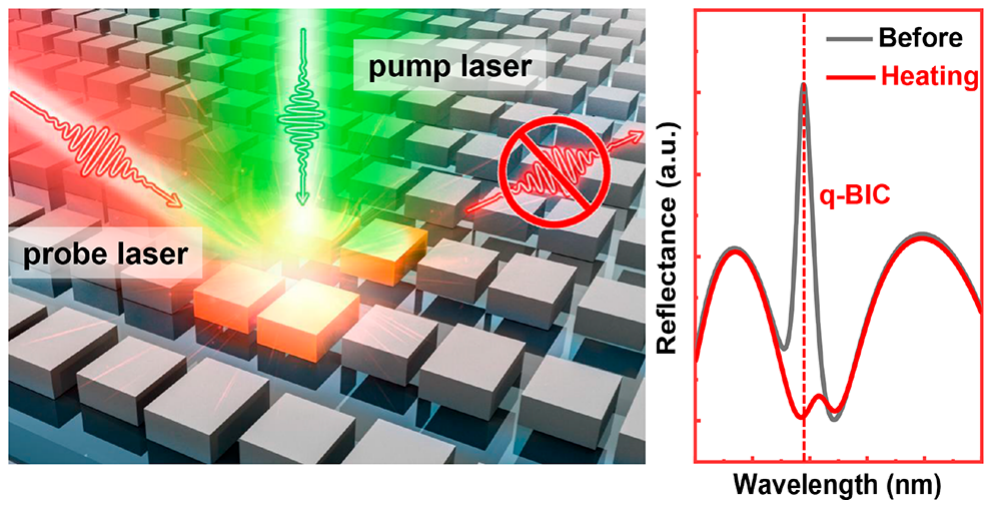

Dynamic access to quasi-bound states in the continuum (q-BICs) offers a highly desired platform for silicon-based active nanophotonic applications, while the prevailing tuning approaches by free carrier injections via an all-optical stimulus are yet limited to THz and infrared ranges and are less effective in visible bands. In this work, we present the realization of active manipulations on q-BICs for nanoscale optical switching in the visible by introducing a local index perturbation through a photothermal mechanism. The sharp q-BIC resonance exhibits an ultrasensitive susceptibility to the complex index perturbation, which can be flexibly fulfilled by optical heating of silicon. Consequently, a mild pump intensity of 1 MW/cm^2^ can yield a modification of the imaginary part of the refractive index of less than 0.05, which effectively suppresses the sharp q-BIC resonances and renders an active modulation depth of reflectance exceeding 80%. Our research might open up an enabling platform for ultrasensitive dynamic nanophotonic devices.

Light confinement, localization, and manipulation in the nanoscale are crucial to realize novel nanophotonic devices with unprecedented performance and functionalities. An emerging field is focused on significantly enhancing the light–matter interactions using subwavelength high-index dielectrics,^1,2^ surpassing the conventional loss limitations of metal-based plasmonics. These all-dielectric nanostructures supporting strong electrical and magnetic Mie resonances play a vital role in the light confinement and give rise to new physical phenomena driven by multipolar interferences and multimodal coupling.^3^ Among them, bound states in the continuum (BICs) have emerged as a new paradigm for trapping and confining the resonant modes. BICs are nonradiating states coexisting with extended waves inside the continuous spectrum range, which have infinitely high quality (Q) factors.^4,5^ Although ideal BICs are considered as dark modes and are decoupled to the free space, small perturbations of the system can transform BICs into quasi-bound states in the continuum (q-BICs) modes with finite but extremely high Q factors, making them accessible to the far field and practically demonstrable for applications. To date, various types of BICs have been realized in diverse photonic systems including photonic crystals,^6^ waveguides,^7^ microcavities,^8^ resonators,^9,10^ and metasurfaces.^11,12^ Featuring high Q factors and ultranarrow bandwidths, optical BICs have unveiled promising potentials in numerous scenarios such as lasing,^13^ nonlinear frequency conversion,^14−18^ chiral enhancement,^12^ narrowband filtering,^19^ and optical sensing and imaging.^20,21^

Beside the general existence and implementations of BICs in nanophotonics in the static form, dynamic access to such BIC and q-BIC modes is highly desirable for active and tunable devices with functionalities on demand. In this respect, incorporating active mechanisms to realize dynamic control of BIC phenomena is increasingly explored. Active modulation of optical spectra and wavefronts of the metasurface based on q-BICs has been demonstrated through global thermal tuning and mechanical stretching^22^ and later via electro-optical modulation^23^ and nanoelectromechanical tuning.^24^ All-optical approaches for reconfigurable q-BIC metasurfaces have garnered intense interest with the assistance of additional active materials such as epsilon-near-zero and phase change materials.^25,26^ Further, ultrafast switching q-BIC metasurfaces have been successfully demonstrated through complex index perturbations via external optical pulses generated by free carriers.^27−29^ However, previous all-optical approaches are mostly limited to BIC modes in the range of terahertz and near-infrared with susceptibility to complex index perturbations.^27,28^ Unfortunately, the interband indirect optical transitions govern the optical losses of silicon and are almost independent of the photodoping level,^30,31^ making the free carrier strategy less effective in visible bands. Thus, practical applications leveraging BICs in the visible light regime for high-contrast switching of radiation still remain challenging.

Here, we introduce a photothermal paradigm that allows dynamic tuning of high-quality q-BIC resonances in broken-symmetry silicon (Si) metasurfaces and demonstrate ultrasensitive optical switching in the visible. The effect of optical heating is elucidated to increase the material loss, thereby annihilating the q-BIC resonances and reducing the Q factor. Leveraging the extreme sensitivity of the sharp resonance of the q-BICs to the material loss, we experimentally reveal the switching of the q-BIC mode to occur at low laser irradiations on the level of ∼MW/cm^2^. It yields a localized temperature increase of around 260 °C corresponding to a modification of the imaginary part of the complex refractive index of less than 0.05, which is sufficient for annihilation of the q-BIC resonance and renders the all-optical modulation strength of reflectance Δ*R*/*R* reaching over 80%. This represents a dramatic improvement over previously reported Si metasurfaces utilizing photothermal nonlinearities which require far higher temperatures for a similar level of modulation.^32−34^ Our results bridge the technology gap in designing BIC-inspired active metamaterials and photonic devices at visible bands and are anticipated to advance active metadevices for various potential applications in modulators, sensors, filters, and dynamic imaging.

The working principle is schematically illustrated in Figure 1a, depicting the all-optical switching of the optical q-BIC. The silicon metasurface is composed of a square lattice of asymmetric silicon bar pairs with different lengths as a unit cell that breaks the in-plane symmetry, which supports a high Q reflective q-BIC mode in the visible. Such a symmetry-protected BIC manifests extreme sensitivity to small perturbations, which is an important prerequisite for switching and modulation. The metasurface is initially illuminated by a probe laser (at 639 nm) for resonant excitation of the q-BIC, thereby producing strong reflection intensity. When additionally imposed by a pump laser (at 532 nm), the metasurface absorbs energy from the incident light, and the complex refractive index of silicon is modified due to the temperature rises, leading to the collapse of the q-BIC mode. Although engineering the complex refractive index contributes to effective all-optical tuning,^35,36^ our following results elucidate that the dynamic amplitude modulation of the q-BIC is not a result of resonance shifting caused by the change of the real part of the refractive index *n* but rather from the increase in the material loss rate mediated by the increase of the imaginary part of the refractive index *k*. By increasing the pump intensity, the q-BIC resonance can be continuously modulated until it is completely quenched, achieving almost 100% switching depth.

**Figure 1:**
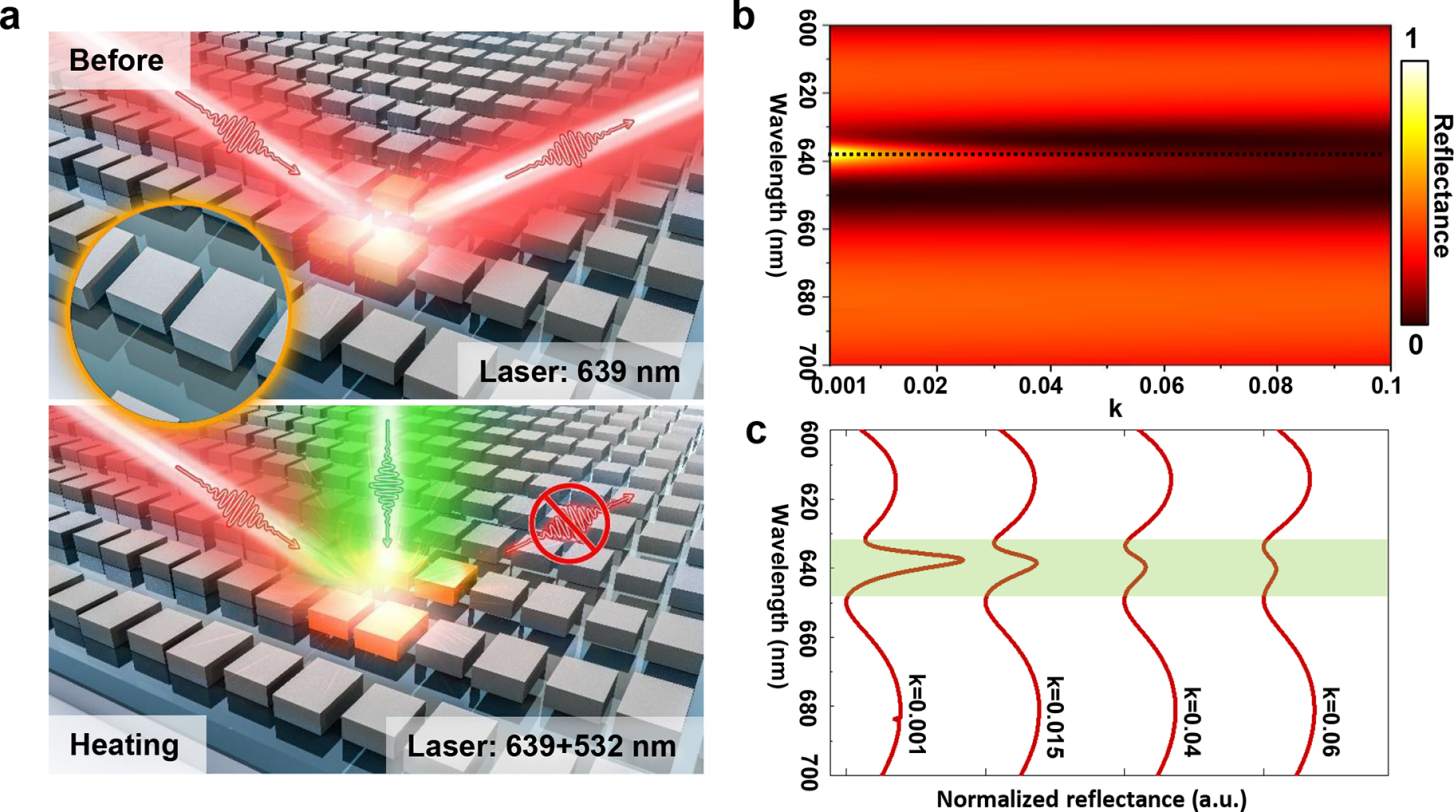
Principle of photothermal control of q-BICs for ultrasensitive nanoscale optical switching. (a) Schematic diagram of photothermal switching of a reflective q-BIC caused by the nonlinear reflection response. (b) Degradation of the q-BIC mode (around 639 nm) with the growth of the imaginary part of the complex refractive index while the real part is fixed at 3.9, which roughly corresponds to parameters of crystalline silicon in the visible range. (c) Four representative reflectance spectra extracted from (b) illustrating the reduction of the reflectance associated with the Q factor of the q-BIC mode at different *k* values.

The susceptibilities of the q-BIC mode to the imaginary part of the refractive index are analyzed theoretically. In the calculations, the real part of the index is fixed at 3.9, which approximately corresponds to parameters of crystalline silicon in the visible range, and the imaginary part is varied. The evolution of the reflection spectra of the metasurface with respect to *k* is demonstrated in Figure 1b, showing that the sharp resonance associated with the q-BIC mode decreases rapidly with a slight changes of *k* while the position of the resonance peak remains almost unchanged. The offset reflection spectra for several representative *k* values plotted in Figure 1c clearly manifest that sophisticated modification of *k* by less than 0.1 serves to increase the material loss of the metasurface and effectively switches off the ultrasensitive sharp resonance mode in terms of broadening of the resonance line width and reduction of the intensity, rendering the large modulation depth approaching 100%. Importantly, neither the resonance wavelength nor the intensities of other resonant modes are susceptible to the modification of *k* except for q-BIC. In the following, we will implement dynamic control of the q-BIC through regular engineering by means of a photothermal mechanism. As *k* can be simply tailored via the pump beam intensity, it thus offers an ideal all-optically controlled and flexible strategy to manipulate the BIC system at will.

Figure 2a shows a sketch of the designed metasurface structure. We consider resonant metasurfaces composed of meta-atoms in the form of an asymmetric pair of rectangular silicon bars on a silicon-on-insulator (SOI) wafer. The bars in each pair have lengths of *b*_1_ and *b*_2_, respectively, and their difference Δ*l* = *b*_1_ – *b*_2_ contributes to the asymmetry of the unit cell. The length *b*_2_ is fixed to be 200 nm while *b*_1_ is varied in the range of 50–400 nm. The metasurface has a square lattice with a period of 400 nm in both the *x* and *y* directions, thickness of 70 nm, bar width of 150 nm, and distance between bars of 50 nm. Figure 2b shows the full map of theoretical reflection spectra by varying the length *b*_1_, in which a normal incident *y*-polarized light excites the collective lattice resonances, and the realistic dispersive property of the crystalline silicon with losses is taken into account. As can be seen in the figure, the broken-symmetry metasurfaces demonstrate sharp resonant responses in the normal incidence reflection associated with q-BICs in the visible wavelength region around 600–700 nm. The q-BIC mode (enclosed by the green dashed lines for eye guidance) undergoes continuous red shifts with increasing bar length *b*_1_. The narrow reflection peak vanishes in the spectrum when the pair of bars becomes symmetric, manifesting the formation of the ideal BIC state (marked by a white circle). Following the resonance evolution trajectory, we extracted the Q factors from the reflection spectra and plotted them in Figure 2c. One can see that the line width of the q-BIC reduces rapidly as the asymmetry Δ*l* approaches zero, such that the Q factor diverges and grows to infinity when the bar pairs have the same length.

**Figure 2:**
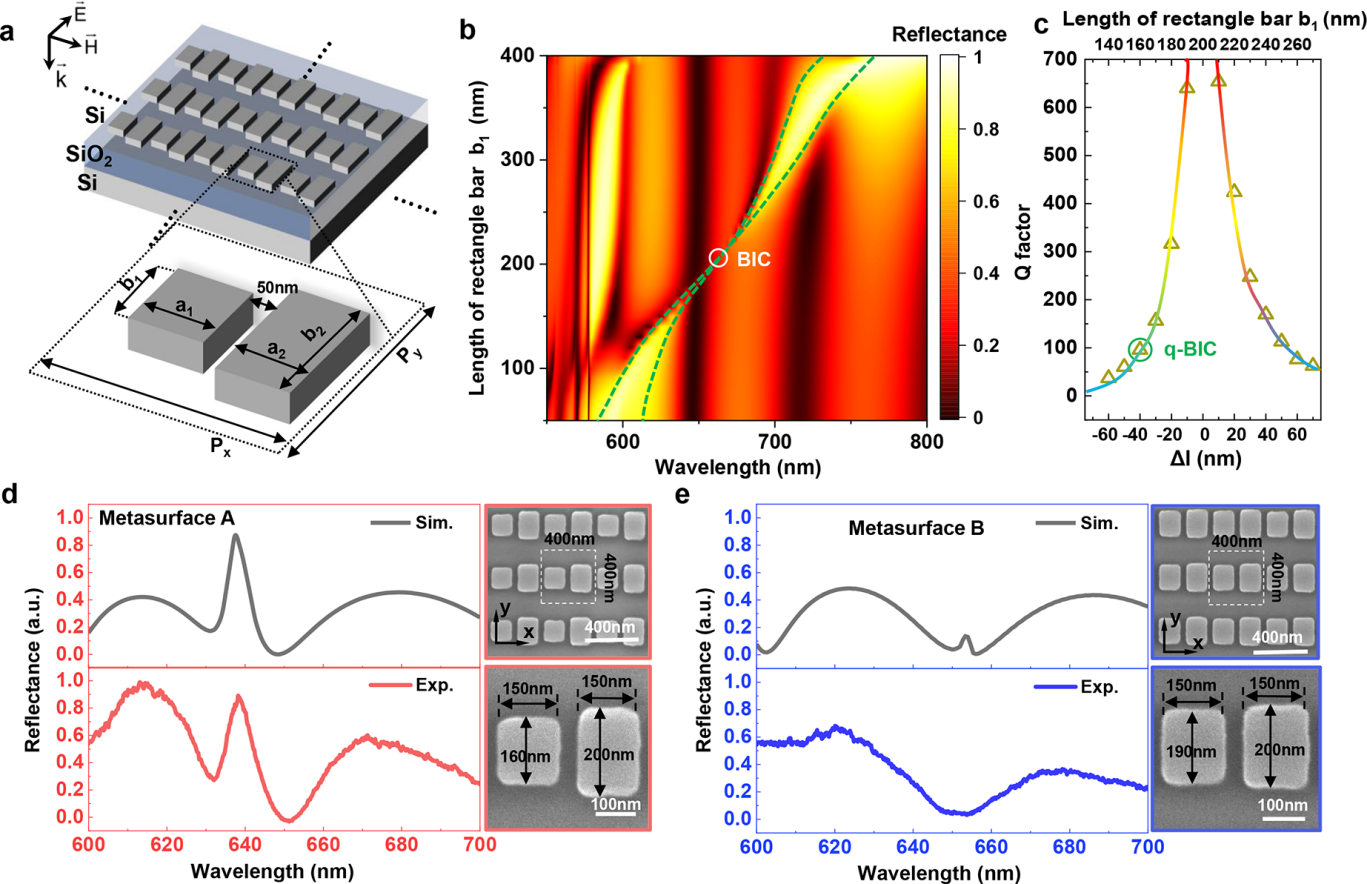
Geometrically tunable high-Q dielectric metasurfaces based on q-BIC modes. (a) Schematic of the all-dielectric metasurface supporting q-BICs. The Si bar pairs are placed on the SOI substrates. Structural parameters: *a*_1_ = *a*_2_ = 150, *b*_2_ = 200, *b*_1_ = 50–400, and *P_x_* = *P_y_* = 400 (units in nanometers). The polarization of the incident light is also indicated. (b) Evolution of the reflection spectra of the metasurface with respect to bar length *b*_1_, showing the q-BIC mode (enclosed by the green dashed lines for eye guidance) undergoes continuous red shifts with increasing the bar length *b*_1_. (c) Dependence of the Q factor of the q-BIC mode on the asymmetry parameter Δ*l*. Experimental and numerical reflectance spectra of metasurface A (d) and metasurface B (e) and the corresponding SEM characterizations.

To validate our designs, we fabricated a set of BIC metasurfaces with length *b*_1_ ranging from 140 to 190 nm. The fabrication was done on a SOI wafer with a 70 nm top silicon device layer on a 2 μm buried oxide. The fabrication of the metasurface was based on the standard electron-beam lithography and reactive-ion etching process, thus being compatible with the scalable complementary metal-oxide–semiconductor (CMOS) process. The scanning electron microscopy (SEM) images of the two fabricated samples are shown in Figure 2d,e, with the specific size parameters indicated. SEM images of the *xz* cross section were also examined to verify the full etching process such that the thickness of the Si meta-atoms was consistent with the thickness of the silicon device layer of the SOI (Figure S1). Experimental measurements of the reflection spectra of both samples are presented, showing excellent agreement with their respective numerical simulations (see Supporting Information Note 2 for more details). Herein, two representative metasurfaces are being considered. For the metasurface A, the bar length *b*_1_ in the unit cell was set to be 160 nm, such that it hosted a sharp q-BIC resonance at a wavelength of 639 nm in the reflection, for the purpose of matching the resonance of the metasurface with the probe laser wavelength for ultrasensitive optical switching. Both simulation and experimental results confirmed the presence of the q-BIC at 639 nm with a Q factor of ∼100. Such a q-BIC mode was also analyzed by utilizing multipole expansions (Figure S2), showing the origin of the dominant contribution from the magnetic dipole (MD) mode and the electric quadrupole (EQ) mode. As a comparison, the metasurface B had *b*_1_ to be 190 nm, corresponding to the resonance wavelength of the q-BIC mode red-shifted away from 639 nm, meaning its off-resonance state under the probe beam excitation. In the following, we show the distinct photothermal switching behaviors of these two samples.

The measurement of the photothermal switching q-BIC state was conducted based on a standard reflection confocal laser-scanning microscope (CLSM), equipped with continuous-wave (CW) laser lines of 532 and 639 nm for confocal excitation (Figure 3a, also see Supporting Information Note 3 for details). Linear polarization excitation was controlled by imposing a half-wave plate on the 639 nm laser beam to ensure the appropriate polarization for q-BIC excitation. Besides, an additional supercontinuum source and the spectrometer were coupled into the microscope to record dynamic reflection spectra from the metasurface during optical pumping by a 532 nm laser. The incident light was focused on the sample surface by an air objective with a numerical aperture (NA) of 0.4, assuring the sufficient number of meta-atoms being illuminated to support collective lattice resonances. A Gaussian focus approximately covered the 5 × 5 unit cells of the metasurface, and the corresponding electromagnetic field distributions at the q-BIC resonance at λ = 639 nm are shown in Figure 3b. This suggests the operating metasurface can be ultracompact with an effective working area of only 2 × 2 μm^2^.

**Figure 3:**
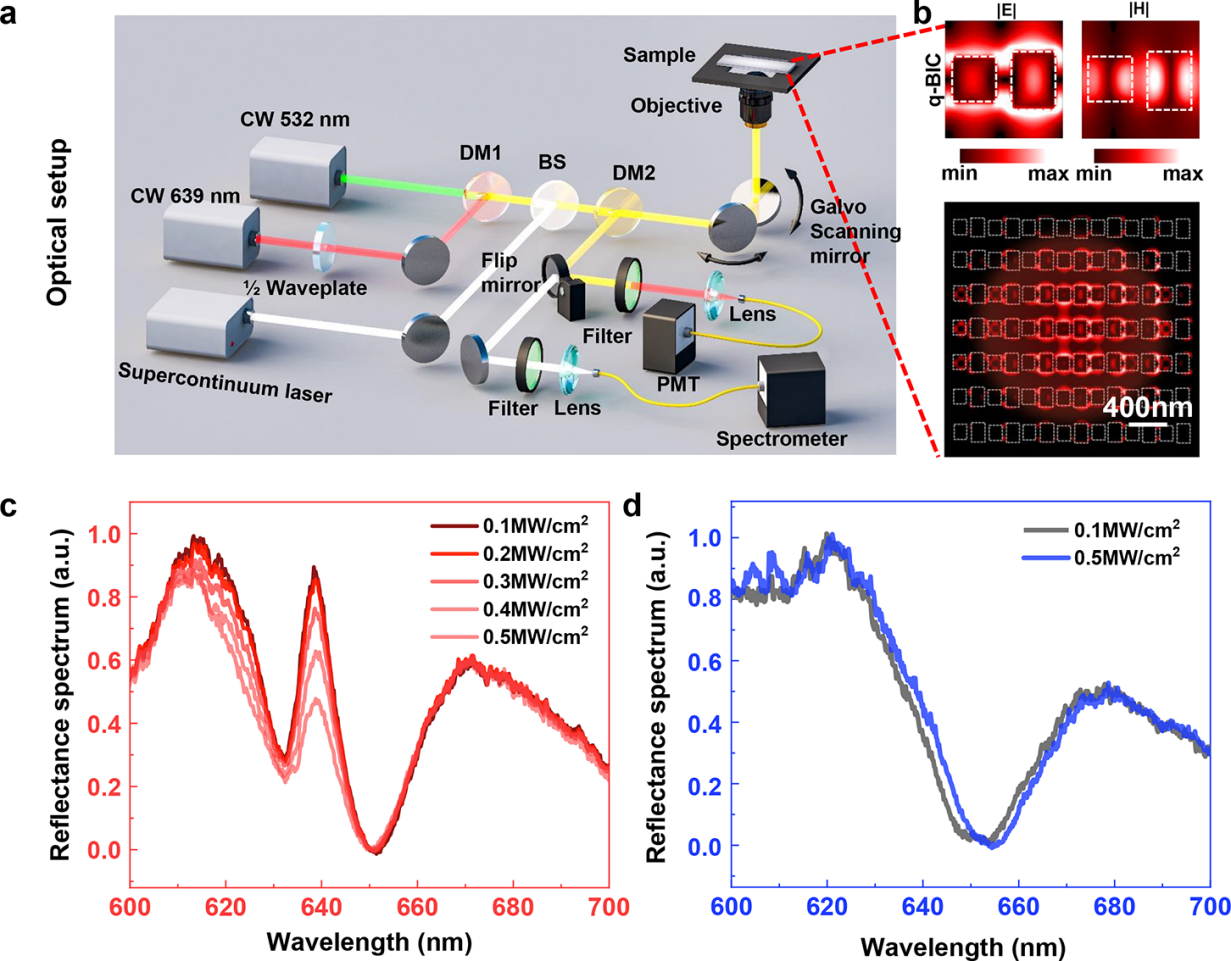
Experimental observations of photothermal switching of the q-BIC mode revealed by dynamic reflection spectra. (a) Optical setup for the photothermal switching measurements. DM, dichroic mirror; BS, beam splitter; PMT, photomultiplier tube. (b) Electromagnetic field distributions of the q-BIC and the one in the center unit cell at a resonance wavelength of 639 nm. (c) Experimentally measured dynamic reflectance spectra of metasurface A (c) and metasurface B (d) with the incident optical power precisely controlled from 0.1 to 0.5 MW/cm^2^.

To identify the photothermal switching q-BIC mechanism, we carried out spectral characterization, allowing the observation of broadband reflection spectra during optical heating by the pump laser. Specifically, we measured the dynamic reflectance spectra at various intensity levels of the 532 nm heating light. Manifested by Figure 3c, the q-BIC mode is strikingly modulated in terms of resonance intensity as well as Q factor, i.e., by increasing the intensity from 0.1 to 0.5 MW/cm^2^, the q-BIC mode annihilation leads to a reflection amplitude reduction reaching 50%. Importantly, it shows that the strong modification occurs only at the q-BIC resonance, while the intensities of routine resonances are minimally affected, meaning that the BIC state is extremely sensitive to the excitation intensity. Improved modulation depth is definitely anticipated with further increases in the pumping intensity. As a sharp contrast, the reflection spectrum of metasurface B exhibits negligible changes when the excitation light intensity is increased accordingly, as shown in Figure 3d.

To further unfold the photothermal mechanism of the q-BIC switching, we link the temperature rise of the metasurfaces with the excitation intensities of the 532 nm laser through Raman thermometry^37^ (Figure S4). The temperatures retrieved from the spectral shift of the Stokes Raman signal show an obvious nonlinear trend. As shown in Figure 4b, the temperature first linearly elevates when the excitation intensity is lower than ∼0.75 MW/cm^2^; then it deviates from the linear slope when the excitation intensity increases over the threshold and rapidly reaches about 285 °C at the excitation intensity of 1 MW/cm^2^.

**Figure 4:**
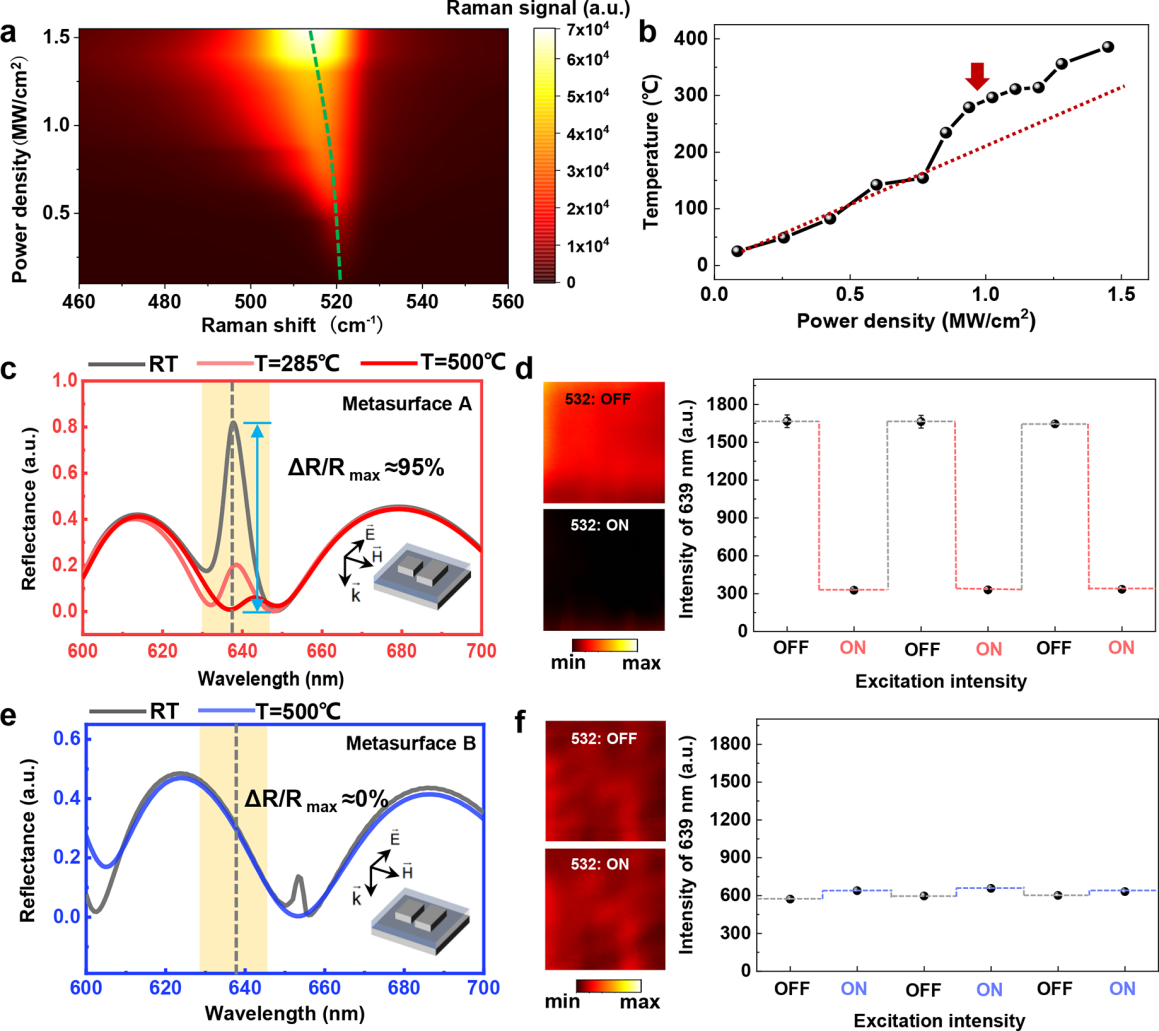
Theoretical and experimental observations of photothermal switching with and without the q-BIC mode. (a) Spectral shift of the Stokes Raman lines of metasurface A irradiated by a 532 nm CW laser at a variety of intensities. (b) Corresponding temperatures retrieved based on Raman thermometry. (c) Theoretical demonstrations of rapid attenuation of the q-BIC mode in metasurface A at three representative temperatures. The susceptibility of q-BIC resonances enables over 80% modulation depth of reflection intensity at a temperature of 285 °C and reaching 95% at a temperature of 500 °C. (d) Experimental observations of reflection modulation in metasurface A. The figures on the left show scanning reflection images obtained with 532 nm pumping light turned on (intensity of 1 MW/cm^2^) or off. The reversibility of switching is confirmed by the full recovery of reflection intensities under multiple measurements. The intensity values recorded before and after the switching (from 1700 to 300, in accordance with Δ*R*/*R* ≈ 82%) indicate that exceeding 80% modulation is achieved. (e,f) For metasurface B, negligible changes of reflection are observed under the same conditions of temperature rises and pumping intensity, manifesting the superiority of the q-BIC mode in ultrasensitive optical switching.

The photoinduced heat generation modifies the refractive index of silicon on both real and imaginary parts, which in turn affects the optical responses of the composing metasurface as a consequence. Therefore, we performed calculations of the reflection spectra by considering the realistic complex refractive index of crystalline silicon at various metasurface temperatures. Figure 4c depicts the simulated reflectance at room temperature (black curve), 285 °C (light red curve), and 500 °C (red curve), where the temperature dependencies of the complex refractive index from crystalline silicon were taken from our previous work for the calculations^33^ (see Figure S5 for details). The reflectance simulated at the elevated temperatures in Figure 4c shows outstanding consistency with the measured ones obtained under the high pumping intensities of the 532 nm laser shown in Figure 3c (see Figure S6 for simulated reflection in a wider range). The ultrasensitive q-BIC mode around 639 nm diminishes over 80% at 285 °C and can almost be further completely switched off, rendering a near-flat spectral response at a temperature approaching 500 °C. It is worth noting that raising the temperature by 260 °C from room temperature results in an increase in the real part of the refractive index *n* by 0.1, while the imaginary part *k* can increase from 0.01 to 0.04 (Figure S5). The impacts on the q-BIC by individual variations in the real and imaginary parts of the refractive index are discussed in Supporting Information Note 7 and compared with these results in Figure 4c when both the real and imaginary index changes are taken into consideration. Although the variation of *n* induces slight resonance shifts up to 2 nm (at 285 °C) and 6 nm (at 500 °C) in the spectrum, the loss by absorption is the dominating factor to quench the strongly resonant amplitudes, leading to the significant suppression of the high-Q mode at mild temperature increases.

Figure 4d demonstrates the all-optical switching leveraging the ultrasensitive metasurface A, where the reflection of the probe beam (at 639 nm) can be efficiently switched off using overlapped excitation of a pump beam (at 532 nm). The active control of reflection resonance is examined through analysis of the reflection images of the metasurfaces. The normalized reflection images are getting darker as the pump intensity increases (more results in Figure S8). More than 80% modulation depth (Δ*R*/*R*) can be readily achieved at the mild pump intensities of 1 MW/cm^2^, corresponding to the realization of the all-optical switch triggered by optical heating under only a slight amount of heating (up to ∼285 °C). To illustrate the superiority of photothermal switching of q-BIC modes, we also performed comparative experiments for another Si metasurface (metasurface B). Metasurface B illuminated at a wavelength of 639 nm corresponds to the condition of off-resonance; therefore, with the same pump intensity of 1 MW/cm^2^, the reflection intensity has almost no modulation (Figure 4e,f). This again consists of the measured broadband spectra in Figure 3d, showing that the overall spectrum remains unchanged during the 532 nm pump excitation.

It should be noted that the exceptional switching capability originates from the huge sensitivity of the reflection high-Q resonant modes, and we exclude the possible variations of optical heating of these two metasurfaces by examinations of their absorption properties (Figure S9). The relevant proof that the two metasurfaces undergo similar temperature elevations is also verified by the Raman measurements. Throughout the process of optical switching, the full recovery of both reflection intensities and corresponding reflection images confirms the excellent reversibility (Figure 4d,f). Note that the heating/cooling effect of silicon nanostructures occurs typically on the order of the nanosecond scale, according to previous research on photothermal tuning.^32,38^ Therefore, the modulation frequency of photothermal switching of the q-BIC state can be anticipated to be up to GHz. Considering that such a nanoscale thermal relaxation time is much faster than the dwell time (10 μs) in our experiments, we measure the reflection intensity after the nanostructures reach thermal equilibrium at each scanning pixel. Further studies of the response time and speed of the photothermal tuning of BIC states are our future goals.

We discuss here that, compared to the previous studies of photothermal nonlinear tuning,^32−34,39−42^ which focus on the strategy of maximizing the absorption to reduce the pump intensity threshold, our work proposes a new scheme that utilizes the high sensitivity of detection signals^31^ for BIC manipulation in the visible band. Generally, it requires large temperature increments to induce pronounced modification of *n* for wavelength tuning, which is a strong perturbation of the system. In this sense, the optical control of BIC modes through a small local index perturbation of *k* that is closely related to the Q factors can be a better candidate. Our study shows that throughout the all-optical manipulations the resonant wavelengths remain well and the slight temperature rise causes an increase in material loss, suppressing the high-Q q-BICs and resulting in a large modulation depth exceeding 80% experimentally. As a final remark, our working principle predicts that a higher Q value serves higher sensitivity of the resonance mode on the imaginary part of the refractive index. In Figure S10, we theoretically reproduce the Si metasurface composing T-shaped meta-atoms to achieve the ultrahigh Q factors in the telecom wavelength region.^16^ The simulation results clearly show that *n* determines the resonance peak position of q-BICs, while *k* dominates the line width and Q value. For the ultrahigh Q system, a slight increase of *k* from 0 to 5 × 10^–3^ enables direct annihilation of the sharp q-BIC resonance peak into a total flat spectrum, i.e., achieving 100% modulation.

In summary, we have investigated the dynamic and substantial manipulation of the q-BIC mode in the visible domain by leveraging its ultrahigh sensitivity to a small photothermal perturbation. We designed and experimentally demonstrated optical q-BICs with Q factors of ∼100 in the visible using a subdiffraction lattice of asymmetric Si nanobar pairs. Temperature measurements show that the metasurface undergoes a temperature increase of ∼260 °C under mild pump intensities of 1 MW/cm^2^ by a 532 nm laser. The temperature elevation induces modification of the imaginary part of the refractive index, which is the dominant factor to directly switch off the q-BIC resonance. The monitoring of broadband full reflection spectra clearly illustrates that the strong suppression of the intensity only occurs at the q-BIC, while the resonant wavelength position does not change obviously. The results support the photothermal tuning mechanism arising from the increase in the material loss. Considering the advantages of high contrast ratio, low power consumption, compact device dimensions (footprint of λ^3^), and ease in integrations, our work suggests a new route toward optical modulators through the photothermal engineering of q-BIC resonances and might benefit advanced applications of nonlocal metasurfaces with multifarious functionalities such as dynamic wavefront shaping.^43^

## Data Availability

The data that support the findings of this study are available from the corresponding authors upon request.

## Supporting Information Available

SEM characterizations of metasurfaces, numerical simulations and the experimentally measured reflection spectra, optical setup for the photothermal switching measurements, optical heating of the metasurface, complex refractive index of silicon at elevated temperatures, dynamic reflectance spectra over the wide wavelength range, individual influence of the real and imaginary part of the refractive index on the q-BIC resonance of metasurface, reflection images of metasurface A, absorption properties of two metasurfaces, and the influence of the imaginary part of the refractive index on the q-BIC resonance for higher Q value metasurfaces (PDF)
