# Supplementary material for: Ultrasensitive Photothermal Switching with Resonant Silicon Metasurfaces at Visible Bands
Source: Nano Lett. 2023 Nov 16;24(2):576–83. doi: 10.1021/acs.nanolett.3c03288 (PMC10798257; doi:10.1021/acs.nanolett.3c03288)
Supplement: Supplementary file 1 — nl3c03288_si_001.pdf [file nl3c03288_si_001.pdf]

Supporting Information

Ultrasensitive photothermal switching with resonant silicon metasurfaces at visible bands

Ying Che[1,#], Tianyue Zhang[2,#,*], Tan Shi[1], Zi-Lan Deng[1], Yaoyu Cao[1], Bai-Ou Guan[1], Xiangping Li[1,*]

[1] Guangdong Provincial Key Laboratory of Optical Fiber Sensing and Communications, Institute of Photonics Technology, Jinan University, Guangzhou, 510632, China.
[2] State Key Laboratory of Information Photonics and Optical Communications & School of Integrated Circuits, Beijing University of Posts and Telecommunications, Beijing 100876, China,
[*] Correspondence: tianyue_zhang@bupt.edu.cn; xiangpingli@jnu.edu.cn
[#]These authors contributed equally to this work.

Supporting Information Note 1: SEM characterizations of metasurfaces.

Figure S1 (a-f) depict the SEM images of a set of BIC metasurfaces that we fabricated with various lengths of b_1, ranging from 140 to 190 nm. Figures S1(g-h) show the SEM images of the xz-plane indicating the height of the bar-pair nanostructures of 70 nm in the metasurfaces.

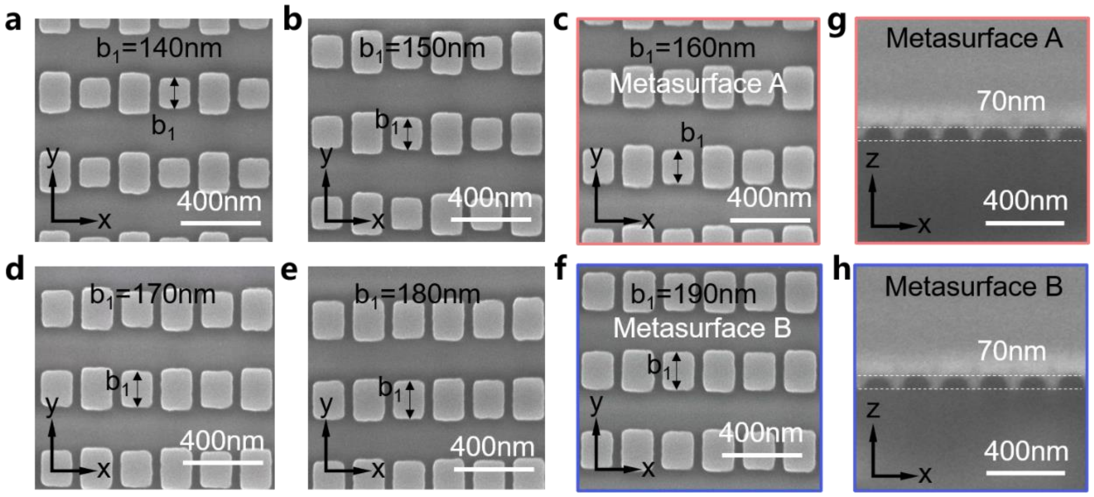

Figure S1. (a-f) xy-plane SEM images of a set of metasurfaces with length b_1 ranging from 140 to 190nm. (g-h) SEM images of metasurfaces A and B in the xz-plane.

Supporting Information Note 2: Numerical simulations and the experimentally measured reflection spectra.

The simulation setup in the FDTD calculations is sketched in Figure S2a. The metasurface sample with 2 µm SiO_2 layer and the silicon substrate were considered in the simulation. The multiple resonances in the reflection spectrum as shown in Figure S2b stem from the complex interactions between Mie resonances of the nanostructures and the Fabry-Perot (F-P) responses of the layered substrate.

In order to elaborate the effect of Mie resonances on the q-BIC, multipolar decompositions of the induced currents in Cartesian coordinates were performed. Figure S2c illustrates that the magnetic dipole (MD) mode and electric quadrupole (EQ) mode primarily contribute to the q-BIC resonance at 639 nm.

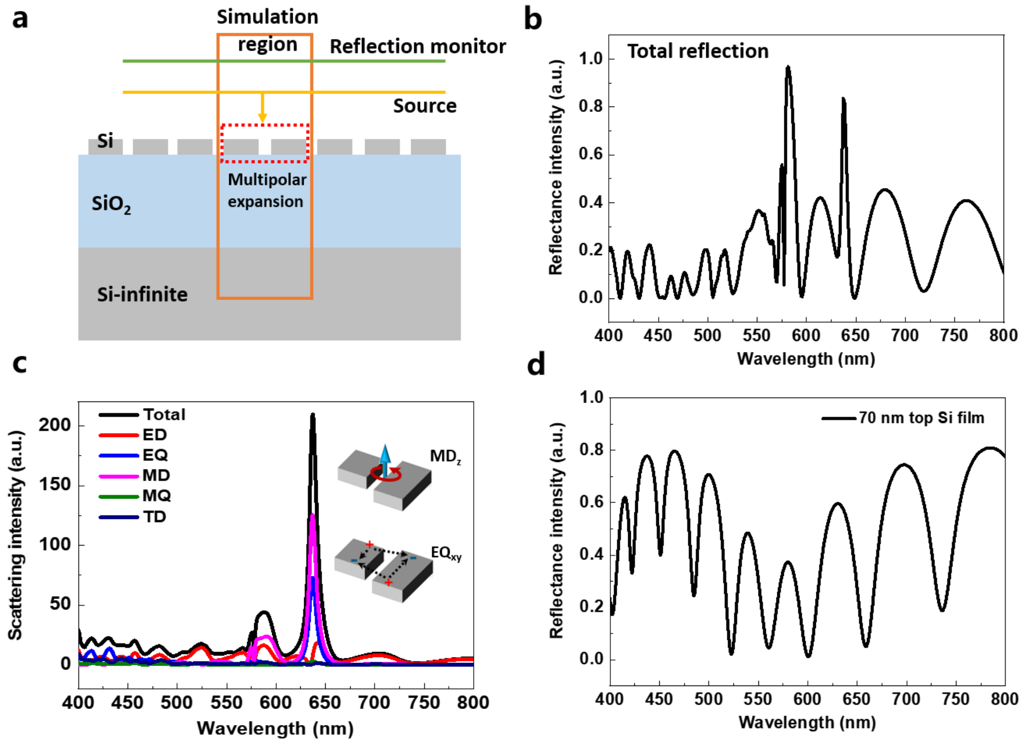

Figure S2. (a) The simulation configuration for FDTD calculations is as follows: The orange frame represents the simulation area, encompassing the x and y span of a single unit cell. To reduce the simulation time, a periodic boundary condition was applied. The yellow line indicates the light source and its incidence direction. A power monitor (the green line) was positioned to record the reflection spectrum. Additionally, box monitors enclosed in red dotted frames were employed to record the electric and magnetic fields for the multipolar decomposition. (b) Simulated reflectance spectrum of metasurface A over the wavelength

range from 400 to 800 nm. (c) Multipolar decomposition of induced currents in Cartesian coordinates. ED, electric dipole; EQ, electric quadrupole; MD, magnetic dipole; MQ, magnetic quadrupole; TD, toroidal dipole. Total is the sum of the Mie scattering contributions of considered multipoles. The insets show the schematic of the multipoles excited at the q-BIC mode. (d) Simulated reflectance spectrum of the layered structure with 70 nm silicon thin film on the top, 2 µm SiO_2 layer in the middle and Si substrate at the bottom, showing the F-P modes originating from the nanocavity.

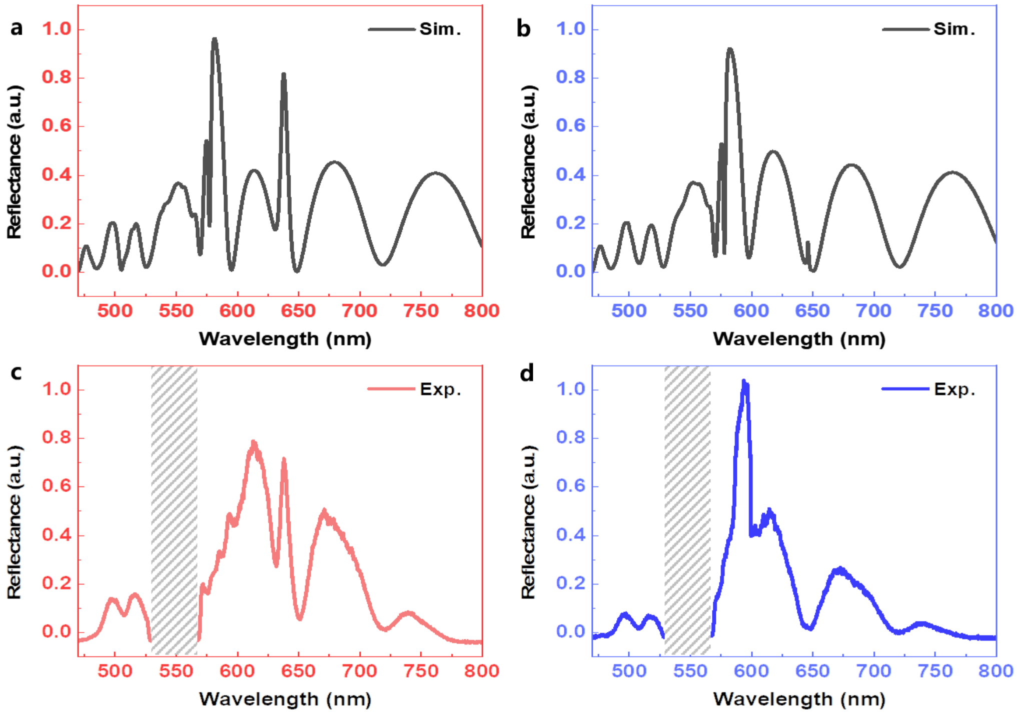

Figure S3. (a-b) Numerical simulations of reflectance spectra of metasurface A (a) and metasurface B (b) over a wide range of wavelength, and the corresponding measured reflection spectra (c-d). The gray shaded areas in (c) and (d) correspond to the unmeasurable parts due to the notch filter used in the spectral measurements.

Supporting Information Note 3: Optical setup for the photothermal switching measurements.

All-optical switch measurements were performed using the set-up depicted on Fig. 3a in the main text, which is based on a reflectance confocal laser scanning microscope. Two CW laser beams (532 nm and 639 nm) and a supercontinuum laser source (OYSL, China) are incorporated into the system coaxially. Both the 639 nm probe laser and supercontinuum laser have sufficiently weak intensity, which are three orders of magnitude lower than the intensity of 532 nm pump laser, such that they would not disturb the absorption of the metasurfaces. An air objective (10×, NA = 0.4, Olympus) was used for both excitation and collection of reflection signals. A half wave plate was used to convert the linearly-polarized 639 nm light into a targeted polarization direction. Dynamic broadband spectral measurements were carried out for verification of the major mechanism of the effective switching. The 532 nm laser with adjustable intensities was used for heating purpose, and the broadband reflection of the metasurface was monitored by a supercontinuum laser, along with a long-pass filter to block 532 nm in front of the Ocean spectrometer (flame-NIR). The switching on and off the q-BIC metasurface were also verified by analyzing the reflection images. A long pass filter was used to spectrally filter the 532 nm pump, leaving only the probe signal to be detected by a photomultiplier tube (PMT) after a confocal pinhole. The scan was performed with a set of galvo-mirrors, and microscope reflection images were obtained by synchronizing the PMT and the galvo mirror scanner and were recorded by beam scanning through the sample with a step size of 7 nm and a dwell time of 10 μs.

Supporting Information Note 4: Optical heating of metasurface.

As Raman scattering is related to optical phonon modes, the shift of the spectral maximum of the crystalline silicon phonon mode (Ω) can be converted into the corresponding temperature shift via the following expression [1]:

$$\Omega(T) = \Omega_0 + \mathrm{A}\left(1 + \frac{2}{e^x - 1}\right) + B(1 + \frac{3}{e^y - 1} + 1 + \frac{3}{(e^y - 1)^2})$$

Where Ω_0 = 528 cm^{-1}, A = −2.96 cm^{-1}, B = −0.174 cm^{-1}, x = $\hbar\Omega_0/2kT$, and y = $\hbar\Omega_0/3kT$ for crystalline silicon. The spectral position of crystalline silicon Raman band is known to experience a blue shift upon heating, such a direct connection between the Raman signal and temperature provides a noncontact way for precise thermometry of the laser-heated structure. The experimentally measured Raman shifts of the metasurface A are shown in Figure S4.

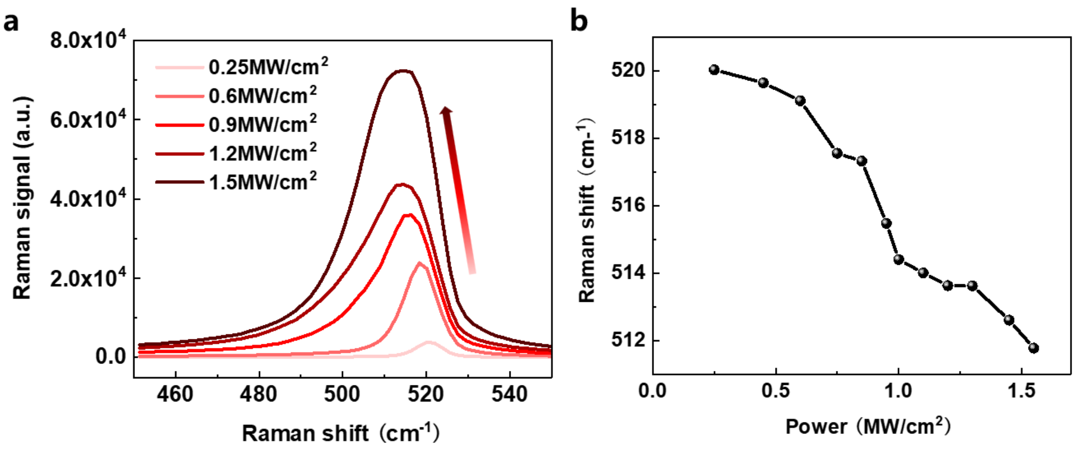

Figure S4. Raman nanothermometry. (a) photothermal-induced shift of the crystalline Si Raman band upon laser heating of the metasurface A under five different irradiance intensities at the wavelength of 532 nm. (b). Raman peak shift as a function of optical power density.

Supporting Information Note 5: Complex refractive index of silicon at elevated temperatures.

We used the refractive index data obtained in our previous work [2] for the calculations in Figure 4c in the main text. Temperature dependence of the complex refractive index of crystalline silicon was measured by using a commercial ellipsometer equipped with a heating stage (M-2000, J.A. Woollam) in the temperature range from 25℃ to 400℃. And the refractive index data above 400℃ was obtained by extrapolation based on the model reported by Jellison and Modine[3].

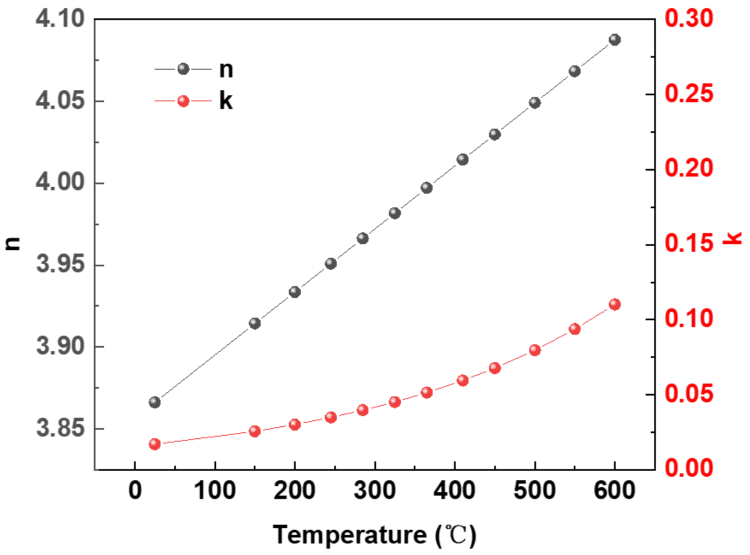

Figure S5. Temperature-dependent real (black) and imaginary (red) refractive index of crystalline silicon at the wavelength of 639 nm from room temperature to 600℃.

Supporting Information Note 6: Dynamic reflectance spectra over the wide wavelength range.

To further compare the reflectance modulations under different resonant modes, we show the dynamic reflectance spectra of metasurface A over a broad range of wavelength. The results in Figure S6 show that the changes in reflection intensity at high temperatures only occur at the wavelength positions corresponding to the Mie resonances (refer to Figure S2c for the locations of Mie resonances), while the F-P modes are negligibly affected. In such content, F-P contributions to the q-BIC switching are ruled out by the fact that F-P modes are primarily depend on the cavity width, thus are far less sensitive to the photothermal effect. In contrary, Mie resonances are more sensitive to changes in the refractive index of the constituent materials. Notably, among all the resonant modes, the q-BIC unambiguously exhibits the most outstanding intensity modulations with increasing the temperature, owing to the ultrasensitivity of the q-BIC to the photothermal perturbation.

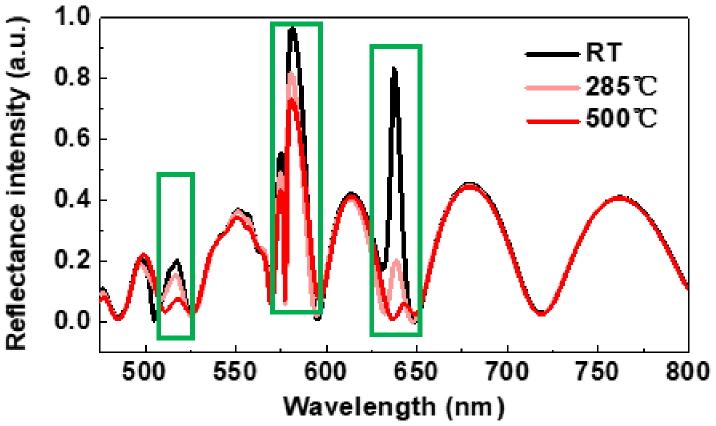

Figure S6. Numerical results of dynamic reflection spectra of metasurface A at different temperatures. The green boxes manifest the positions of Mie resonances with intensity modulations in the presence of temperature elevations under the laser heating. The green boxes highlight the locations of Mie resonances which exhibit observable reflection modulations at elevated temperatures.

Supporting Information Note 7: Individual influence of the real and imaginary part of refractive index on q-BIC resonance of metasurface.

In Fig. 1 in the main text, we perform the simulations of reflectance with different values of the imaginary part k of the refractive index, while keeping the real part n fixed. Here, we also present the results with varying n while keeping k unchanged. As can be seen in Figure S7, a small variation in the real part can induce the resonance shift. Therefore, a large modulation depth can be achieved by either increasing n or k , which is attributed to the fact that q-BIC mode is featured by high Q factors and ultranarrow bandwidth, and the excitation laser delicately operates in the q-BIC mode. However, in real situations, the real and the imaginary parts are varied simultaneously as the temperature increases, hence, the spectrum experiences amplitude reduction along with spectral red-shift. By comparing Figure S7 with Figure 3(c) and Figure 4(c) in the main text, it is clear that the perturbation of k contributes dominantly to the q-BIC resonance manipulation.

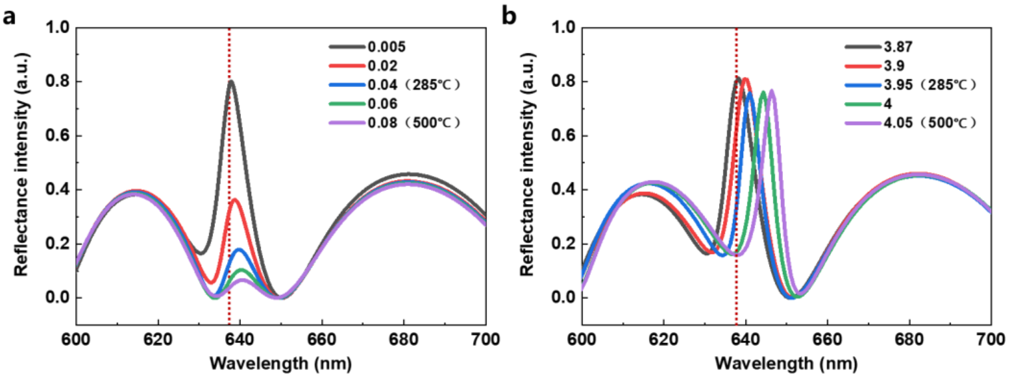

Figure S7. (a) Reflectance spectra of the metasurface A with different values of the imaginary refractive index k, while the real part n is fixed to be 3.87. (b) Reflectance spectra corresponding to different values of the real refractive index n, while the imaginary part k is fixed to be 0.005.

Supporting Information Note 8: Reflection images of metasurface A.

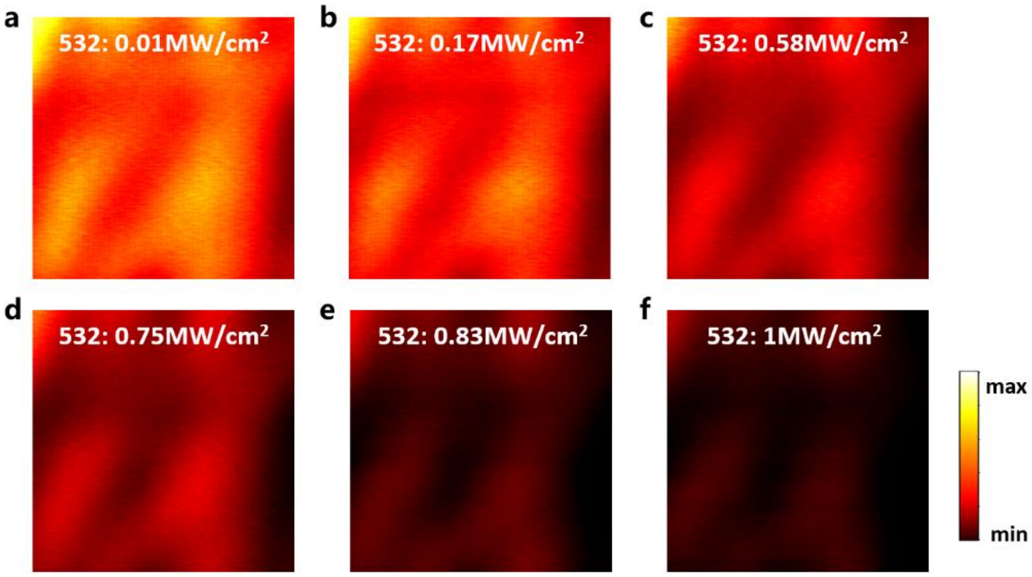

Figure S8. Measured reflection images of metasurface A evolve with increasing pumping intensities of 532 nm laser beam. (a-f) The intensity of 639 nm probe beam is kept constant at 0.01 MW/cm^2, and the different scanning reflection images are acquired at different pumping intensities of 532 nm laser beam.

Supporting Information Note 9: Absorption properties of two metasurfaces.

We performed the calculations of absorption spectra of the two metasurfaces using FDTD for the purpose of comparing their absorption properties. Given that the metasurface designed on the SOI substrate can be treated as a single-port model without transmission, the absorbance can be directly obtained by subtracting the reflectance from 1 (i.e., A=1-R). With the exception of the q-BIC resonance region, the absorption efficiency of both metasurfaces is virtually identical across the whole wavelength band, indicating that the two metasurfaces undergo basically the same temperature elevation under the same excitation conditions.

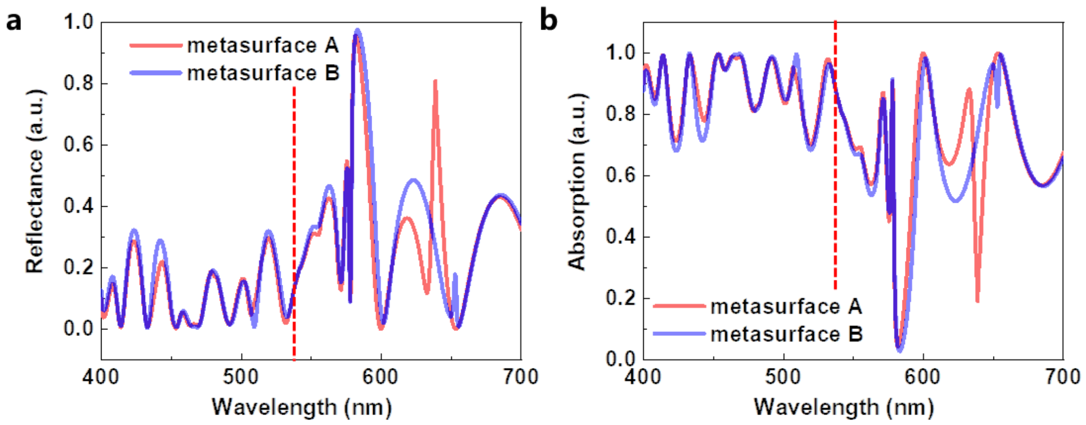

Figure S9. Reflection spectra (a) and absorption spectra (b) of metasurface A and metasurface B. The dashed lines in the figure correspond to the position of the 532 nm, which is the laser utilized to heat the metasurfaces.

Supporting Information Note 10: Influence of imaginary part of refractive index on q-BIC resonance for higher Q value metasurfaces.

In the main text, we demonstrate the proof-of-principle optical switching in the visible region leveraging q-BIC with Q factors ~100. The sensitivity of the photothermal switching of q-BIC system can be remarkably improved with increasing sharpness of the metasurface resonance. We validate this idea by considering Si metasurfaces composed of cuboid nanostructures[4], which support q-BIC mode with ultrahigh Q value of 10^4 in the telecom wavelength region. Specifically, we utilized the regular changes in real part n and the imaginary part k of the refractive index of silicon materials to introduce perturbations to the system and manipulate the q-BIC resonance (Figure S10 a and b). With the increase of n, the corresponding q-BIC resonance redshifts accordingly. In contrast, slight increase of the imaginary refractive index k by 0.005 is sufficient to completely annihilate the sharp q-BIC resonance.

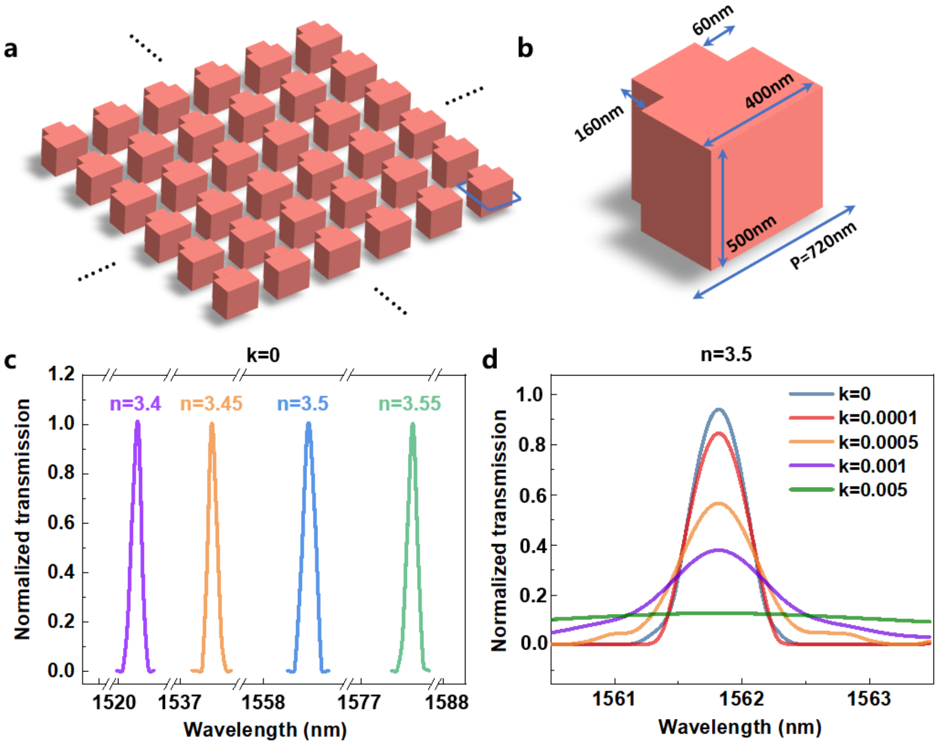

Figure S10. Metasurface supporting ultrahigh q-BICs (a) Periodic arrangement of T-shaped meta-atoms supporting BICs. (b) The structural parameters of the Si blocks. (c) Normalized transmission spectra of the metasurface with different values of the real refractive index n, while the imaginary part k is fixed to be zero. (d) Normalized transmission spectra with different values of the imaginary refractive index k, while the real part n is fixed at 3.5.

Supporting Information References

1. Zograf, G. P.; Petrov, M. I.; Zuev, D. A.; Dmitriev, P. A.; Milichko, V. A.; Makarov, S. V.; Belov, P. A., Resonant Nonplasmonic Nanoparticles for Efficient Temperature-Feedback Optical Heating. *Nano letters* **2017,** *17* (5), 2945-2952.
2. Zhang, T.; Che, Y.; Chen, K.; Xu, J.; Xu, Y.; Wen, T.; Lu, G.; Liu, X.; Wang, B.; Xu, X.; Duh, Y.-S.; Tang, Y.-L.; Han, J.; Cao, Y.; Guan, B.-O.; Chu, S.-W.; Li, X., Anapole mediated giant photothermal nonlinearity in nanostructured silicon. *Nat. Commun.* **2020,** *11* (1) 3027.
3. Jr., G. E. J.; Modine, F. A., Optical functions of silicon at elevated temperatures. *Journal of Applied Physics* **1994,** *76* (6), 3758-3761.
4. Liu, Z.; Xu, Y.; Lin, Y.; Xiang, J.; Feng, T.; Cao, Q.; Li, J.; Lan, S.; Liu, J., High-*Q* Quasibound States in the Continuum for Nonlinear Metasurfaces. *Phys. Rev. Lett.* **2019,** *123* (25), 253901.
